# Obesity and Its Association with Undiagnosed Diabetes Mellitus, High Blood Pressure and Hypercholesterolemia in the Malaysian Adult Population: A National Cross-Sectional Study Using NHMS Data

**DOI:** 10.3390/ijerph20043058

**Published:** 2023-02-09

**Authors:** Hui Chin Koo, Lay Kim Tan, Geok Pei Lim, Chee Cheong Kee, Mohd Azahadi Omar

**Affiliations:** 1 Faculty of Applied Sciences, Tunku Abdul Rahman University of Management and Technology, Jalan Genting Kelang, Kuala Lumpur 53300, Malaysia; 2 Sector for Biostatistics & Data Repository, Office of NIH Manager, National Institutes of Health, Ministry of Health Malaysia, Shah Alam 40170, Selangor, Malaysia; 3 Jeffrey Cheah School of Medicine and Health Sciences, Monash University Malaysia, Bandar Sunway, Subang Jaya 47500, Selangor, Malaysia

**Keywords:** Malaysia, obesity, undiagnosed diabetes mellitus, undiagnosed high blood pressure, undiagnosed hypercholesterolemia

## Abstract

This study aimed to report the prevalence of obesity, classified using Asian cut-off, and its relationships with undiagnosed diabetes mellitus, high blood pressure, and hypercholesteremia. We analyzed the nationally representative data from 14,025 Malaysian adults who participated in the NHMS 2015. The relationship between obesity and undiagnosed diabetes mellitus, high blood pressure, and hypercholesteremia was determined using multivariable logistic regressions, and lifestyle risk factors and sociodemographic characteristics were adjusted. The undiagnosed high blood pressure group showed the highest proportionate of overweight/obese (80.0%, 95% CI: 78.1–81.8) and central obesity (61.8%, 95% CI: 59.3–64.2). Inverse association was observed between underweight with undiagnosed high blood pressure (aOR: 0.40, 95% CI: 0.26–0.61) and hypercholesterolemia (aOR: 0.75, 95% CI: 0.59–0.95) groups. In contrast, positive relationships were shown between overweight/obese and risk of undiagnosed diabetes mellitus (aOR: 1.65, 95% CI: 1.31–2.07), high blood pressure (aOR: 3.08, 95% CI: 2.60–3.63), and hypercholesterolemia (aOR: 1.37, 95% CI: 1.22–1.53). Likewise, central obesity was positively associated with a risk of undiagnosed diabetes mellitus (aOR: 1.40, 95% CI: 1.17–1.67), high blood pressure (aOR: 2.83, 95% CI: 2.45–3.26), and hypercholesterolemia (aOR: 1.26, 95% CI: 1.12–1.42). Our findings indicated the importance of periodical health examinations to assess the risk of non-communicable diseases among the general and abdominal obese Malaysian adults.

## 1. Introduction

Overweight and obesity, in a common term, can be defined with respect to a condition of disproportion between energy consumption and expenditure, which causes unnecessary fat accumulation and results in a number of adverse health effects [1]. This scenario involves a complex interaction between the metabolic and genetic on one hand, and lifestyle and socio-cultural factors on the other hand [2]. The prevalence of obesity is increasing tremendously due to the combination of both unhealthy eating and sedentary lifestyle [3]. It has been underlined as a worldwide health issue by the World Health Organization (WHO). Globally, obesity prevalence has observed a triple escalation since 1975, whereby in 2016, more than 1.9 billion overweight cases were seen in the adult population, of these over 650 million were obese, affecting approximately 2 billion people’s health [4]. This scenario is not only observed in developed countries, but is happening in developing countries too. Among the eleven countries in Southeast Asia, Malaysia reported the highest obesity prevalence in the adult population [5]. An approximately five-fold rise in obesity prevalence has been reported in Malaysia in the last two decades, from 4.4% in 1996 [6] to 19.7% in 2019 [7]. If the global trends continue as they are, about 1 billion adults will be obese, whereas 2.7 billion adults will be overweight; over 177 million adults will be severely affected by obesity by 2025 [4].

The detrimental effects of overweight and obesity on health have been well recognized. Outcomes of a longitudinal study showed that obesity has elevated the risk of mortality and aggravated non-communicable diseases (NCDs) [8]. Typically, a minimum of 25 kg/m^2^ body mass index (BMI) will increase the risk of getting NCDs. In spite of that, the WHO also proposed a minimum of 23 kg/m^2^ BMI should be applied in Asian populations [9]. The outcomes from the National Health and Morbidity Survey (NHMS) reported a high percentage of undiagnosed NCDs, suggesting that health screening might not be happening at lower BMI thresholds [10]. According to a meta-analysis, BMI has commonly been the selected measurement by which to determine nutritional status. Nevertheless, other alternative measurements that reflect central obesity, e.g., waist–hip ratio (WHR), are also being proposed as surrogate indicators of “hidden fat”; it is a greater indicator to predict NCDs, e.g., cardiovascular diseases [11]. Due to their low cost and ease of measurement, these anthropometric measurements are applied in epidemiological studies for population surveillance of risk factors for NCDs [12].

Metabolic syndrome is characterized as a set of metabolic abnormalities including cardiovascular disease, high blood pressure, and insulin resistance. A systematic review has shown a positive relationship between obesity and metabolic syndrome [13]. However, the relationship between both variables is still ambiguous. Metabolic syndrome can occur in normal-weight individuals [14], and not all obese individuals have metabolic syndrome [15]. Studies have shown a significant association between metabolic syndrome and oxidative stress [16]. Undiagnosed metabolic syndrome is a serious public health threat as these cases remained untreated and are subsequently at a higher risk of developing severe NCD-relevant complications [17]. A high prevalence of undiagnosed NCDs in Malaysia may lead to increasing the cumulative burden of NCDs in the country and contribute to the rise in healthcare costs in Malaysia [7]. Understanding the major risk factor of undiagnosed NCDs is vital to mitigate the significant growth in the burden of NCDs. Overweight and obesity is one of the key drivers of the global rise in NCDs. However, the time interval between screenings and the age to begin screening for obese individuals has not been well defined [18]. This is an importance issue, in the view that NCDs lead to a substantial mortality and morbidity prevalence, which can be alleviated through early diagnosis and treatment with weight loss being a key management goal [19].

Governments and policymakers are tending to pay more attention to the population diagnosed with NCDs, while lesser attention has been given to the importance of identifying the major risk factors for at-risk individuals to manage undiagnosed NCDs. Nonetheless, recognizing the major risk factor among individuals at risk for NCDs is becoming imperative to reduce the onset of NCDs. Limited studies have reported on the association between obesity and undiagnosed NCDs, and thus much remains unknown about specific risk factors associated with undiagnosed NCDs. Therefore, this paper aims to describe the obesity prevalence using a nationwide representative dataset. We also present the associations between obesity and undiagnosed diabetes mellitus, high blood pressure, and hypercholesterolemia. To our knowledge, the present paper is the first to associate overweight and obesity with undiagnosed diabetes mellitus, high blood pressure, and hypercholesterolemia among the Malaysian population. The present paper may give insight into the associations between obesity and undiagnosed diabetes mellitus, high blood pressure, and hypercholesterolemia for the government and policymakers and give and insight into combatting obesity and NCDs in Malaysia.

## 2. Materials and Methods

### 2.1. Study Design and Sampling

In this present study, we used the data from the National Health and Morbidity Survey (NHMS) 2015 to investigate the relationship between obesity and undiagnosed diabetes mellitus, high blood pressure, and hypercholesterolemia [10]. Briefly, the NHMS 2015 was a population-based cross-sectional study (i.e., a nationally representative dataset of non-institutionalized Malaysian population) to determine the morbidity, health status, and health care demands among Malaysian adults aged 18 years and above. Those staying in hotels, hostels, hospitals, and others were grouped as an institutional population and hence these individuals were excluded from the NHMS 2015 survey. The general objective of the NHMS 2015 was to measure health-related community-based data and information that will aid the Ministry of Health Malaysia in reviewing, evaluating, and setting its health priorities, program strategies, and activities, and planning its allocation of resources [10].

To ensure the collected data were nationally representative, the respondents for NHMS 2015 were selected by means of stratified two-stage proportionate-to-size cluster sampling design. The stratified two-stage proportionate-to-size cluster sampling design comprised the primary and secondary stratums. In the primary stratum, all the thirteen states and three Federal Territories were included. Meanwhile, the secondary stratum comprised the urban and rural areas in each state that was involved. The Department of Statistics Malaysia (DOSM) provided the 2014 updated sampling frame for the sampling process. The sampling frame was the list of the geographical areas in Malaysia that were divided into enumeration blocks (EBs). The EBs were classified into either urban or rural areas, based on the population size of the gazette area in 2014. Urban area was defined as a gazette area that has a combined population of 10,000 or more, whilst rural area was a gazette area that has a population of less than 10,000 individuals. There were approximately 75,000 EBs in Malaysia in the year 2014, in which each EB comprises 80 to 120 living quarters (LQs). Each LQ comprises an average population of 500 to 600 individuals. The probability-proportional-to-size sampling technique was employed, where a total of 10,428 LQs were selected from the total EBs in Malaysia. Of these 10,428 LQs, 536 EBs were randomly selected from the urban areas, and 333 EBs from the rural areas. During the survey, all the households identified within the selected LQs were included and all the members in each of the identified household were eligible and were invited to participate in the survey. The detailed methodology and sampling design of NHMS 2015 were described elsewhere [10].

### 2.2. Ethical Consideration

The ethical approval from the Medical Research and Ethics Committee, Ministry of Health Malaysia was obtained (NMRR-14-1064-21877) prior to the commencement of the national health and morbidity survey. Following the ethical obligation, all the eligible respondents were informed about the nature of the NHMS 2015 by the trained enumerators. Then, written informed consent from the eligible respondents who agreed to participate was obtained prior to the interviews.

### 2.3. Survey Materials and Data Collection

The detailed survey materials and data collection of NHMS 2015 were published elsewhere [10]. In a nutshell, data collection using the standardized pre-validated structured questionnaires was performed between March and June 2015. The modes of data collection were either face-to-face interviews using the e-NHMS 2015 application programmed in a mobile device by trained enumerators or self-administered by participants using hardcopy questionnaires.

Next, the trained nurses collected the anthropometry data (i.e., weight, height, and waist circumferences) [20], as well as measured the blood pressure [18] and fasting blood glucose and cholesterol levels [21] of the respondents. The fasting blood glucose and cholesterol levels of the respondents were assessed using the capillary finger-stick blood sampling methods. The weight (kgs) and height (cm) of each respondent was measured with duplicate readings using the validated Tanita Personal Scale HD319 and SECA Stadiometer 213. For field implementation purposes, a standard weight was supplied to the trained nurses for standardization during the weight measuring process. Then, the blood pressure [22] was measured three times using the validated and calibrated Omron Japan Model HEM-907 machine. To assess the level of fasting blood glucose and cholesterol, the validated PA Cardiocheck was used, where only one reading for the respective fasting blood glucose and cholesterol levels was taken.

### 2.4. Study Variables

#### 2.4.1. Independent Variables

The independent variables included in this present study were general obesity and central obesity that were classified based on the body mass index (BMI) value and waist circumference value, respectively. The BMI value in each respondent was calculated as the body weight (kg) divided by square of heights (m^2^). In this present study, the Asia-Pacific classification for obesity was applied for the classification of both general obesity and central obesity. Hence, based on the Asia-Pacific classification, the general obesity status of the respondents was classified into three groups, i.e., (i) underweight (<18.5), (ii) normal (18.5–22.9), and (iii) overweight/obese (>23) [20]. As for central obesity, a respondent with waist circumference value above 90 cm in men or 80 cm in women was defined as abdominally obese [20].

#### 2.4.2. Dependent Variables

The dependent variables included in this study were undiagnosed diabetes mellitus, undiagnosed high blood pressure, and undiagnosed hypercholesterolemia. Undiagnosed diabetes mellitus was defined as a respondent who self-reported non-diabetes mellitus and had a fasting capillary blood glucose of 6.1 mmol/L or more (or non-fasting blood glucose of more than 11.1 mmol/L) at the time of recruitment [23]. Meanwhile, undiagnosed high blood pressure was defined as a respondent who self-reported non-high blood pressure and had a systolic blood pressure of 140 mmHg or more and/or a diastolic blood pressure of 90 mmHg or more [18]. Lastly, undiagnosed hypercholesterolemia was defined as a respondent who self-reported non-hypercholesterolemia and had a total blood cholesterol of 5.2 mmol/L or more [21]. Furthermore, the respondents with undiagnosed diabetes mellitus, undiagnosed high blood pressure, and/or undiagnosed hypercholesterolemia self-reported not receiving treatments for diabetes mellitus, high blood pressure, and/or hypercholesterolemia in the past one year at the time of recruitment. In the regression model, whenever one of the health conditions was defined as a dependent variable, the other two health conditions were included as covariates (i.e., confounding factors) in the model. For example, when undiagnosed diabetes mellitus was defined as a dependent variable, undiagnosed high blood pressure and undiagnosed hypercholesterolemia were included in the model as covariates.

#### 2.4.3. Potential Confounders

We included two categories of covariates—(i) the sociodemographic characteristics and (ii) lifestyle risk factors—in the regression model. For the sociodemographic characteristics, we included sex, age, ethnicity, residential area, marital status, educational level, and monthly household income as covariates, whilst adequate fruit and vegetable intake, alcohol consumption, smoking status, and physical activity were covariates for the lifestyle risk factors category. Furthermore, the participants’ age was categorized into five different age groups as follows: (i) 18–29 years, (ii) 30–39 years, (iii) 40–49 years, (iv) 50–59 years, and (v) 60 years and above. The respondents self-reported their ethnicity status during recruitment and were classified as Malay, Chinese, Indian, other *Bumiputera* and others, accordingly. Based on the local Malaysian education system, the education level of the respondents was classified as follows: (i) no formal education, (ii) primary education, (iii) secondary education, and (iv) tertiary education.

The Malaysian household income classification published by the Department of Statistics Malaysia was used for classification of the total monthly household income and the categories were as follows: (i) bottom 40% (B40) with monthly income below RM 4850 (approximately USD 1084), middle 40% (M40) with monthly income between RM 4851 and RM 10,970 (approximately USD 1084–USD 2452), and top 20% (T20) with monthly income above RM 10,971 (above USD 2452). We reported the monthly household incomes in USD currency from here onwards.

A respondent was classified of having adequate daily fruit and vegetable intake when more than five servings (more than 3 servings of vegetable and 2 servings of fruit) were consumed based on the Malaysian Dietitian Guidelines 2020, otherwise inadequate [24]. The alcohol consumption status of the respondents was categorized as never and ever (i.e., current and former drinker). Meanwhile, the smoking status of the respondents was categorized as never, current smoker, and former smoker. The short version of the International Physical Activity Questionnaire was used as the instrument to assess the physical activity level data of the respondents for the NHMS 2015 [10], where the physical activity of the respondents was categorized as inactive and active [25].

We assessed the quality of this present study using the checklist adapted from STrengthening the Reporting of OBservational studies in Epidemiology (STROBE) specifically for cross-sectional study (Supplementary Table S1).

### 2.5. Statistical Analyses

A total of 19,959 Malaysian adults aged 18 years old and above participated in the NHMS 2015 [10]. In this present study, we focused primarily on obesity and its relationship with undiagnosed diabetes mellitus, undiagnosed high blood pressure, and undiagnosed hypercholesterolemia. Hence, participants with unclear/unknown citizenship status, medically diagnosed with diabetes mellitus, high blood pressure and hypercholesterolemia, and missing data about their health conditions were removed from the subsequent analyses. Briefly, we removed individuals who were (i) non-Malaysians/unknown citizenships (*n* = 1285), (ii) medically diagnosed with diabetes mellitus, high blood pressure, and hypercholesterolemia (*n* = 4639), and (iii) missing data about their health conditions (*n* = 10). For these reasons, the final number of individuals that were included in the subsequent data analyses was 14,025. Of these, 1567, 3276, and 6554 individuals were not known to have diabetes mellitus, high blood pressure, and hypercholesterolemia, respectively.

Firstly, we performed the complex samples descriptive statistics to illustrate the sociodemographic characteristics, lifestyle risk factors, and health status (i.e., undiagnosed diabetes mellitus, high blood pressure, and hypercholesterolemia) of the respondents, stratified by obesity status. Then, the complex samples multivariable logistic regression was performed to investigate the relationship between obesity (i.e., general obesity and central obesity) and the risk of undiagnosed diabetes mellitus, undiagnosed high blood pressure, and undiagnosed hypercholesterolemia. In this present study, a total of 1001 and 1047 individuals out of 14,025 individuals had missing data for the respective general obesity and central obesity. Hence, both the descriptive analysis and the multivariable logistic regression with complex sampling design were restricted only to those study subjects with no missing data of the general obesity and central obesity status. Then, we determined the adjusted odd ratios (aOR) with the respective 95% confidence intervals (95% CIs) to measure the association between general obesity/central obesity and undiagnosed diabetes mellitus, undiagnosed high blood pressure, and undiagnosed hypercholesterolemia. A *p*-value of less than 0.05 indicates a statistically significant association. As the complex sample analyses were employed, the sample in this present study was weighted and hence the findings from this study represent the general population of Malaysian adults aged 18 years and above. Lastly, we evaluated the model fit and predictive ability of the final model using the respective pseudo-R-squares value, classification tables, and receiver operating characteristics (ROC) analyses. The IBM Statistical Package of Social Sciences (SPSS) for Windows version 27.0 (IBM Corp., Armonk, NY, USA) was used to perform all the above-mentioned statistical analyses.

## 3. Results

The sociodemographic characteristics, lifestyle risk factors, and health status of the respondents in this study were presented in Table 1. Our descriptive analysis showed that the mean age (years old, ±SE) of the respondents was 36.77 (±0.18) and the proportion of the male and female respondents were comparable (male: 51.4%; female: 48.6%). The majority of the respondents resided in an urban area (76.3%), were married (60.4%), had secondary level education (51.5%), with a monthly household income less than USD 1084, and worked in the private sector (47.5%).

We defined obesity as a lifestyle factor as this health condition developed gradually as a result of poor dietary patterns and lifestyle choices (e.g., exceeded daily intake of free sugar, eating large amounts of processed or fast food, sedentary lifestyle, overconsumption of alcohol drinks, and smoking). Classification of obesity status among the respondents using BMI value demonstrated that 60.9% of the respondents were overweight/obese, whilst 41.9% were obese when using waist circumference for obesity classification. Our data further demonstrated that 85.4% of the total respondents were non-drinkers, followed by 76.1% and 67.7% of the total respondents who were never smokers and physically active, respectively. Only 2.8% of the total respondents had an adequate daily intake of fruit and vegetable. As for health status, our data showed that undiagnosed hypercholesterolemia had the significantly highest proportion of total respondents, i.e., 42.6% (95% CI: 41.3–44.0), followed by undiagnosed high blood pressure (proportion: 19.4%, 95% CI: 18.5–20.4), and undiagnosed diabetes mellitus (proportion: 96%, 95% CI: 8.8–10.4).

**Figure 1:**
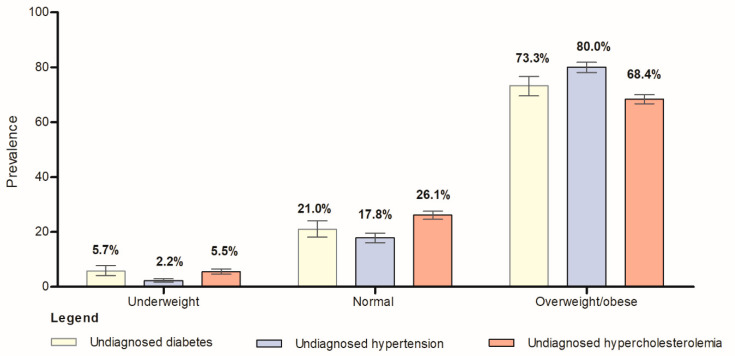
The prevalence of undiagnosed diabetes mellitus, undiagnosed high blood pressure, and undiagnosed hypercholesterolemia stratified by general obesity status among the Malaysian adults (*n* = 13,024).

We further analyzed the characteristics of the respondents stratified by obesity status (i.e., general obesity and central obesity). Table 2 shows the characteristics of the 13,024 respondents stratified by general obesity status (i.e., normal, underweight, and overweight). The Pearson’s chi-square test demonstrated that there were significant associations between age group (*p* < 0.0001), ethnicity (*p* < 0.0001), marital status (*p* < 0.0001), education level (*p* < 0.01), occupation (*p* < 0.0001), daily fruit and vegetable intake (*p* = 0.04), smoking (*p* < 0.01), and physical activity (*p* < 0.0001) with overweight/obese among the Malaysian adults. There was a significantly higher prevalence rate of overweight/obese among the individuals who were aged between 40 and 49 years old (72.1%, 95% CI: 69.4–74.6), of Indian origins (72.1%, 95% CI: 67.7–76.2), were married (68.5%, 95% CI: 67.1–69.8%), had primary level education (62.8%, 95% CI: 60.0–65.6), worked in the government sector (72.1%, 95% CI: 68.8–75.2), had adequate daily intake of fruit and vegetables (69.0%, 95% CI: 62.8–74.7), were former smokers (70.6, 95% CI: 61.6–78.3), and were physically active (63.0, 95% CI: 61.6–64.5). We further observed a significantly higher prevalence of overweight/obese in individuals with undiagnosed diabetes mellitus (73.3%, 95% CI: 69.6–76.7), undiagnosed high blood pressure (80.0%, 95% CI: 78.1–81.8), and undiagnosed hypercholesterolemia (68.4%, 95% CI: 66.7–70.0) in this present study (Figure 1, Supplementary Table S2).

**Figure 2:**
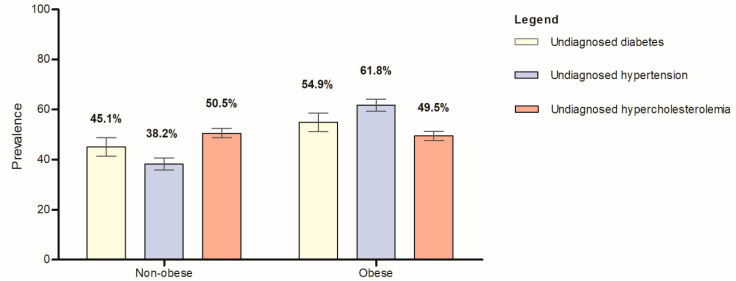
The prevalence of undiagnosed diabetes mellitus, undiagnosed high blood pressure, and undiagnosed hypercholesterolemia stratified by central obesity status among the Malaysian adults (*n* = 12,978).

Table 3 presents the characteristics of 12,978 respondents stratified by central obesity status (i.e., non-obese and obese). We observed significant associations between sex (*p* < 0.0001), age groups (*p* < 0.0001), ethnicity (*p* < 0.0001), marital status (*p* < 0.0001), educational level (*p* < 0.01), occupation (*p* < 0.0001), fruit and vegetable intake (*p* < 0.01), alcohol intake (*p* < 0.01), and smoking status (*p* < 0.0001) with central obesity among the Malaysian adults. Our data demonstrated a higher prevalence of central obesity among the individuals who (i) were women (52.2%, 95% CI: 50.4–54.1), were aged between 50 and 59 years old (53.5%, 95% CI: 50.3–56.6), (ii) were Indians (59.7%, 95% CI: 54.8–64.4), (iii) were widow/widower/divorcee (53.4, 95% CI: 49.2–57.5), (v) had primary education (46.8%, 95% CI: 44.1–49.6), (iv) were unpaid/homemakers (59%, 95% CI: 56.0–62.0), (v) had adequate daily intake of fruit and vegetables (51.3%, 95% CI: 45.1–57.4), (vi) were non-drinkers (42.7%, 95% CI: 41.3–44.1), and (vii) were never-smokers (45.8, 95% CI: 44.2–47.4). Similar to the obesity classification by BMI value, we observed a significantly higher prevalence of central obesity in individuals with undiagnosed diabetes mellitus (54.9%, 95% CI: 51.2–58.6), undiagnosed high blood pressure (61.8%, 95% CI: 59.3–64.2), and undiagnosed hypercholesterolemia (49.5%, 95% CI: 47.6–51.3) in this present study (Figure 2, Supplementary Table S3).

**Figure 3:**
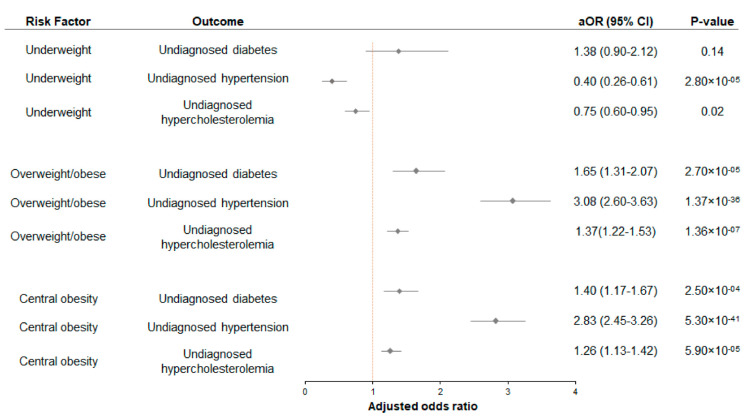
Associations between general obesity and central adiposity with undiagnosed diabetes mellitus, undiagnosed high blood pressure, and undiagnosed hypercholesterolemia in Malaysian adults aged 18 years and above. Multiple logistic regression was performed, and adjusted for sociodemographic (i.e., sex, age, ethnicity, residential area, marital status, educational level, household monthly income) and lifestyle risk factors (i.e., adequate fruit and vegetable intake, alcohol intake, smoking status, physical activity). There were no significant interactions among the independent variables. aOR: adjusted odds ratio; 95% CI: 95% confidence interval.

The association analysis between general obesity and health status demonstrated that underweight was associated negatively with undiagnosed high blood pressure (aOR: 0.40, 95% CI: 0.26–0.61, *p*: 2.80 × 10^−05^) and undiagnosed hypercholesterolemia (aOR:0.75, 95% CI: 0.59–0.95, *p* = 0.02), after adjustment for sociodemographic characteristics, lifestyle factors, and health conditions (Figure 3). Our data further demonstrated that overweight/obese was associated with an increased risk of undiagnosed diabetes mellitus (aOR: 1.65, 95% CI: 1.31–2.07, *p*: 2.70 × 10^−05^), undiagnosed high blood pressure (aOR: 3.08, 95% CI: 2.60–3.63, *p*: 1.37 × 10^−36^), and undiagnosed hypercholesterolemia (aOR: 1.37, 95% CI: 1.22–1.53, *p*: 1.36 × 10^−07^) after adjustment for sociodemographic characteristics, lifestyle factors, and health conditions (Figure 3). The evaluation of the model fit showed low variation between the expected and observed data of the final model for undiagnosed diabetes, undiagnosed hypertension, and undiagnosed hypercholesterolemia groups with the respective pseudo-R-square values (Nagelkerke’s R squares) of 0.051, 0.145, and 0.096 (Supplementary Table S4). Then, the predictive ability of the final model showed that the final models were fit, where the correct predictive values from the classification tables were 89.7%, 80.7%, and 62.5% for the respective undiagnosed diabetes mellitus, undiagnosed high blood pressure, and undiagnosed hypercholesterolemia (Supplementary Table S4). Then, the ROC analysis demonstrated that the value of the area under curve (AUC) for the respective undiagnosed diabetes mellitus, undiagnosed high blood pressure, and undiagnosed hypercholesterolemia groups were 0.66 (95% CI: 0.65–0.68), 0.75 (95% CI: 0.74–0.76), and 0.67 (95% CI: 0.66–0.68) (Supplementary Table S4).

We further observed that central obesity was associated with an increased risk of undiagnosed diabetes mellitus (aOR: 1.40, 95% CI: 1.17–1.67, *p*: 2.51 × 10^−04^), undiagnosed high blood pressure (aOR: 2.83, 95% CI: 2.45–3.26, *p*: 5.28 × 10^−41^), and undiagnosed hypercholesterolemia (aOR: 1.26, 95% CI: 1.12–1.42, *p*: 5.90 × 10^−05^) (Figure 3). The evaluation of the model fit showed low variation between the expected and observed data of the final model for undiagnosed diabetes, undiagnosed hypertension, and undiagnosed hypercholesterolemia groups with the respective pseudo-R-square values (Nagelkerke’s R squares) of 0.049, 0.140, and 0.090 (Supplementary Table S4). The predictive ability of the final model assessment showed high correct predictive values from the classification tables, which were 89.7%, 80.5%, and 62.0% for the respective undiagnosed diabetes mellitus, undiagnosed high blood pressure, and undiagnosed hypercholesterolemia (Supplementary Table S4). The AUC obtained from the ROC analysis for the respective undiagnosed diabetes mellitus, undiagnosed high blood pressure, and undiagnosed hypercholesterolemia groups were 0.66 (95% CI: 0.64–0.68), 0.75 (95% CI: 0.74–0.76), and 0.67 (95% CI: 0.66–0.68) (Supplementary Table S4).

## 4. Discussion

To our knowledge, this is the first paper to report on the associations between obesity and undiagnosed diabetes mellitus, high blood pressure, and hypercholesterolemia from a nationwide representative dataset. High prevalence of obesity (i.e., general and central obesity) was observed in the present study, among those who were unaware of having diabetes mellitus, high blood pressure, and hypercholesterolemia among the Malaysian adults. We further reported the significant associations between obesity and undiagnosed diabetes mellitus, high blood pressure, and hypercholesterolemia. A high cumulative prevalence of overweight/obesity was presented in our study, which was associated with a high rate of undiagnosed diabetes mellitus, high blood pressure, and hypercholesterolemia in the Malaysian population. Since a number of diseases have been associated with obesity, understanding the complications of obesity is imperative for developing successful prevention interventions.

We found that nearly 6 and 4 in 10 Malaysians had general and central obesity, respectively. For the past several years, obesity prevalence has increased tremendously in most of the Southeast Asian countries, and Malaysia has the dubious honor of having the highest proportion of obese and overweight citizens in the region [4]. This national epidemic was especially predominant among the Indian community [26]. The outcomes of our study complement a previous study from Malaysia, which revealed that obesity prevalence among Indians was the highest in 17,257 adults aged more than 17 years [27]. Several studies have discussed the varieties of cultural practices and dietary patterns as potential reasons for higher obesity rates in the Indian population [28]. Our finding of a greater prevalence of obesity in people living in urban areas is similar to a nationally representative survey from Myanmar [29]. Urbanization is always correlated with dramatic changes in dietary patterns and improvements in public transportations, facilities and infrastructures in urban zones [29]. Furthermore, social media prominently advertising messages to increase the consumption of processed foods, sugary drinks, and fast foods in urban regions may lead to the accessibility and availability of food which is high in sugar, fat, and carbohydrates [30]. The present study also showed that obesity was more prevalent in the married and primary-level-educated community. Similarly, a study from Taiwan demonstrated a negative correlation between educational status and obesity in 59,927 adults [31]. A number of possible aspects should be considered for elucidating this relationship. The highly educated community may have greater awareness of the complications of obesity and may have healthier lifestyles [5]. Therefore, at the academic level we should note this when establishing obesity-related interventions or policies.

Surprisingly, the present study showed the higher obesity prevalence among the individuals who consumed an adequate intake of fruit and vegetables. Our finding opposed the present conception of the favorable wellbeing effects of fruit and vegetable intake [32,33,34]. However, a narrative review concluded that the supporting indication for intake of fruit and vegetables in weight management is still inconclusive [35]. This outcome of the present study is in accordance with the data published by Shi et al. (2011) [36], who reported that “vegetable-rich” dietary habits caused weight gain in their longitudinal study. Perhaps “unnoticeable” fatty ingredients included in vegetable preparation and the types of vegetable, e.g., starchy vegetables, should be taken into consideration when drawing conclusions. Our point of view is supported by a review which mentioned that starchy vegetables, e.g., corn, potatoes, pumpkins, and peas, were positively correlated with weight elevation, and the higher glycemic load could explain this relationship [32]. Another study also demonstrated a more favorable impact on body weight management from fruit intake than vegetable consumption [37]. Thus, fruit and vegetable intake should be measured individually when it comes to the weight change investigation. The possible mechanism of fruit and vegetable consumption in obesity development may be illustrated in a future study [38]. Furthermore, our outcome also yielded contrary findings with an increased obesity prevalence. The positive effect of exercise on weight management is complicated and possibly reciprocal. Substantial evidence has demonstrated the inverse association between weight management and physical activity [39]. Yet, a review revealed a number of discrepancies in the literature to conclude this association [40]. Understanding the complex of obesity is imperative and the varied strengths of exercise might contribute to various outcomes on weight management.

Obesity is well known as a prominent risk factor for several non-communicable diseases (NCDs), those with obesity also account for a high prevalence of undiagnosed diabetes mellitus, high blood pressure, and hypercholesterolemia in the present study. Our findings are consistent with the National Health and Nutrition Examination Survey (1988–2020) from the US, where overweight or obese adults had the highest undiagnosed diabetes mellitus prevalence [41]. Similarly, previous studies from Malaysia [42] and Sudan [43] indicated that obese adults have significantly higher chances of having undiagnosed high blood pressure; further, obese Sudanese had the highest prevalence of undiagnosed high blood pressure. Hence, addressing obesity is the predominant strategy in reducing the prevalence of undiagnosed NCDs. Our findings relevant to obesity raise an important public health concern. Even more alarming is the finding that overweight and obese plays a vital part in the high prevalence of undiagnosed diabetes mellitus, high blood pressure, and hypercholesterolemia. The relationship between obesity and undiagnosed NCDs can be explained by several potential mechanisms. In one such theoretical mechanism, the substances which are involved in the development of diabetes mellitus, including non-esterified fatty acids, proinflammatory markers, hormones, glycerol, and cytokines, will increase among overweight/obese adults [44]. The theoretical mechanisms with relation to obesity and high blood pressure include renin-angiotensin-aldosterone system stimulation, alteration of the functional and structural kidney, changes in adipose-derived cytokines, and sympathetic nervous system overaction [45].

Obesity, undiagnosed diabetes mellitus, high blood pressure, and hypercholesterolemia are largely preventable. Changes in lifestyle can reduce both body weight and undiagnosed NCDs [41]. It is crucial to establish appropriate care that may decline morbidity and mortality on early detection of the diseases, often when the person is essentially asymptomatic. However, the age to begin screening and the time interval between screenings for overweight and obese adults has not been well established at the time of this survey. Early detection of diseases is a vital initial step to manage the diseases and their related morbidity and mortality, given that diabetes mellitus, high blood pressure, and hypercholesterolemia can be silent for years and mitigated through early detection and treatment, with weight loss as the major key management goal [46]. Early detection may also gain some indirect advantages, specifically for overweight and obese adults, by encouraging them to lose their body weight and start a healthy way of life. Healthy lifestyle has been demonstrated to decline the development of NCDs [41]. Further research is recommended to investigate the public health effect of screening approaches that incorporate a nuanced consideration for overweight and obesity in the future.

### Strengths, Limitations, and Future Recommendations

Our study has some strengths worth noting. The nationwide representative big sample size and the stratified inclusion of study respondents via a home-to-home visit approached by healthcare professionals makes the study unique, and the outcomes are crucial to monitor the health of the Malaysian population. Furthermore, our analyses only included the Malaysian adult population who were unaware of having diabetes mellitus, high blood pressure, and hypercholesterolemia; we have excluded those were confirmed with diabetes mellitus, high blood pressure, and hypercholesterolemia. In addition, our study has adjusted lifestyle risk factors and sociodemographic characteristics to eliminate the potential bias.

Although the findings are promising, they have a number of limitations that need to be acknowledged. One such limitation is that this was a cross-sectional study; hence, our outcome could only reflect the association between obesity and undiagnosed diabetes mellitus, high blood pressure, and hypercholesterolemia without presenting the chronological order. Moreover, the data were mainly based on self-reporting, which may result in some biased results. Nonetheless, self-administered questionnaires were applied in the present survey, which may reduce some of the bias effects. A single reading of fasting blood glucose was taken in this national study; hence, the outcome may not be able to identify the trend of the fasting blood sugar level. Lastly, the cholesterol measurements in the study participants using the capillary blood from finger-prick method may not be able to estimate the true value of blood cholesterol.

Taken all into account, a longitudinal study may be recommended for future research to determine the causal relationship between obesity and undiagnosed diabetes mellitus, high blood pressure, and hypercholesterolemia in the Malaysian adult population. Future studies might also include the venous blood to measure the blood glucose and cholesterol, as venous blood probably provides a more accurate reading compared to capillary blood from finger-pricks.

## 5. Conclusions

On the whole, a high prevalence of overweight/obesity was observed in the Malaysian adult population. Positive associations have been demonstrated between obesity with undiagnosed diabetes mellitus, high blood pressure, and hypercholesterolemia. Our findings may give insight to the government and policymakers, and further enhance the current health education program. The outcome of the present study gives insight to the Ministry of Health to emphasize the importance of periodic health examinations and regular health screenings in all the populations. Moreover, the risk of undiagnosed diabetes mellitus, high blood pressure, and hypercholesterolemia in the Malaysian population, specifically those with general and central obesity issues, should be highlighted in the policy or healthcare program.

## Figures and Tables

**Table 1:** Characteristics of the Malaysian adult respondents aged 18 years and above (*n* = 14,025).

Characteristic	Estimated Population	Count (*n*)	(%)	95% CI
***Sociodemographic***				
**Sex**				
Male	7,349,538	6802	51.4	50.3–52.4
Female	6,961,477	7223	48.6	47.6–49.7
**Age group (years old)**				
18–29	5,618,744	4336	39.3	37.9–40.6
30–39	3,506,540	3214	24.5	23.3–25.8
40–49	2,376,196	2567	16.6	15.7–17.6
50–59	1,614,064	2098	11.3	10.6–12.0
≥60	1,195,471	1810	8.4	7.8–9.0
**Ethnicity**				
Malay	7,820,984	9256	54.7	51.7–57.6
Chinese	3,644,065	2318	25.5	22.8–28.3
Indian	968,769	972	6.8	5.8–7.9
Other Bumiputera	1,665,962	1280	11.6	10.1–13.4
Others	211,236	199	1.5	1.0–2.1
**Residential area**				
Urban	10,920,733	8041	76.3	75.1–77.5
Rural	3,390,282	5984	23.7	22.5–24.9
**Marital status**				
Single	4,902,331	3703	34.3	32.9–35.7
Married	8,646,849	9265	60.4	59.0–61.8
Widow/widower/divorcee	761,834	1057	5.3	4.9–5.8
**Education level**				
No formal education	514,075	675	3.6	3.2–4.1
Primary	2,108,803	2637	14.9	14.0–15.8
Secondary	7,295,664	7054	51.5	50.2–52.9
Tertiary	4,238,517	3543	29.9	28.4–31.5
**Monthly household income**				
B40	9,637,764	10,051	67.3	65.3–69.3
M40	3,641,622	3222	25.4	23.9–27.1
T20	1,031,629	752	7.2	6.0–8.6
**Occupation**				
Government	1,477,554	1698	12.3	11.2–13.5
Private	5,702,054	4518	47.5	45.9–49.1
Self-employed	2,625,292	2946	21.9	20.6–23.1
Unpaid/homemaker	1,902,359	2158	15.8	15.0–16.7
Retiree	295,486	381	2.5	2.1–2.8
***Lifestyle factors***				
**General Obesity**				
Underweight	1,085,293	945	8.2	7.5–8.9
Normal	4,097,610	3722	30.9	29.8–32.0
Overweight/Obese	8,075,208	8357	60.9	59.7–62.1
**Central adiposity**				
Normal	7,660,972	7056	58.1	56.7–59.4
Obese	5,534,879	5922	41.9	40.6–43.3
**Alcohol intake**				
Never	12,205,147	12,602	85.4	83.8–86.9
Ever	2,084,719	1406	14.6	13.1–16.2
**Smoking**				
Never	10,880,437	10,773	76.1	74.9–77.2
Current	3,217,163	3041	22.5	21.4–23.6
Former	206,763	205	1.4	1.2–1.7
**Physical activity**				
Inactive	4,561,762	4257	32.3	31.1–33.5
Active	9,559,315	9582	67.7	66.5–68.9
**Daily fruit and vegetable**				
Inadequate	13,825,446	13,531	97.2	96.8–97.6
Adequate	391,066	407	2.8	2.4–3.2
***Health status***				
Undiagnosed diabetes mellitus	1,370,631	1567	9.6	8.8–10.4
Undiagnosed high blood pressure	2,780,088	3276	19.4	18.5–20.4
Undiagnosed hypercholesterolemia	6,102,589	6554	42.6	41.3–44.0

Data source from National Health and Morbidity Survey (NHMS) 2015; B40: bottom 40% (household income below USD 1084); M40: middle 40% (household income between USD 1084 and USD 2452); T20: top 20% (household income above USD 2452); 95% CI: 95% confidence interval.

**Table 2:** Characteristics of the Malaysian adult respondents aged 18 years and above stratified by general obesity status (*n* = 13,024).

Characteristic	Estimated Population	Count (*n*)	Underweight (*n* = 945)		Normal (*n* = 3722)		Overweight/Obese (*n* = 8357)		*p*-Value *
			Prevalence (%)	95% CI	Prevalence (%)	95% CI	Prevalence (%)	95% CI	
***Sociodemographic***									
**Sex**									0.09
Male	6,913,170	6406	7.6	6.7–8.6	30.5	28.9–32.1	61.9	60.2–63.6	
Female	6,344,941	6618	8.8	7.8–10.0	31.4	29.8–33.0	59.8	58.1–61.5	
**Age groups (years)**									<0.0001
18–29	5,191,917	3983	13.4	12.0–14.9	37.7	35.7–39.6	49.0	46.9–51.0	
30–39	3,196,043	2933	5.0	4.0–6.1	26.9	24.8–29.2	68.1	65.8–70.3	
40–49	2,275,513	2465	4.4	3.3–5.7	23.6	21.4–25.9	72.1	69.4–74.6	
50–59	1,513,664	1993	2.7	1.9–3.9	26.7	24.1–29.4	70.6	67.8–73.3	
≥60	1,080,974	1650	8.5	6.7–10.7	31.6	28.8–34.6	59.9	56.6–63.1	
**Ethnicity**									<0.0001
Malay	7,235,399	8591	7.9	7.1–8.8	29.1	27.9–30.3	63.0	61.6–64.3	
Chinese	3,311,338	2110	10.1	8.3–12.1	37.8	35.2–40.6	52.1	49.1–55.1	
Indian	903,031	906	6.2	4.1–9.1	21.7	18.2–25.7	72.1	67.7–76.2	
Other Bumiputera	1,603,952	1224	7.1	5.4–9.1	29.5	26.1–33.0	63.5	59.6–67.2	
Others	204,391	193	4.7	2.2–9.8	34.8	27.0–43.6	60.4	51.6–68.6	
**Residential area**									0.22
Urban	10,068,977	7439	8.1	7.2–9.0	30.5	29.2–31.8	61.4	59.9–62.9	
Rural	3,189,134	5585	8.5	7.5–9.6	32.3	30.3–34.3	59.2	57.1–61.3	
**Marital status**									<0.0001
Single	4,623,681	3492	14.2	12.6–15.9	37.9	35.8–40.0	47.9	45.7–50.2	
Married	7,942,613	8565	4.9	4.3–5.5	26.6	25.4–27.9	68.5	67.1–69.8	
Widow/widower/divorcee	691,817	967	6.4	4.6–8.8	33.2	29.3–37.3	60.4	56.3–64.4	
**Education level**									<0.01
No formal education	448,826	598	10.8	7.9–14.6	33.2	28.4–38.5	56.0	50.6–61.3	
Primary	1,972,388	2488	5.8	4.6–7.2	31.4	28.8–34.1	62.8	60.0–65.6	
Secondary	6,813,524	6582	9.2	8.2–10.4	30.2	28.7–31.7	60.6	58.9–62.3	
Tertiary	3,890,678	3258	7.3	6.2–8.6	31.6	29.5–33.8	61.1	58.7–63.4	
**Monthly household income**									0.07
B40	8,938,318	9333	8.6	7.8–9.5	31.0	29.7–32.3	60.5	59.0–61.9	
M40	3,366,294	2990	8.1	6.8–9.6	30.4	28.0–32.8	61.5	58.7–64.3	
T20	953,499	701	4.7	3.2–6.8	32.3	28.2–36.8	63.0	58.5–67.3	
**Occupation**									<0.0001
Government	1,379,375	1581	4.7	3.5–6.4	23.1	20.3–26.3	72.1	68.8–75.2	
Private	5,275,335	4199	7.9	6.9–9.1	32.6	30.6–34.6	59.5	57.5–61.5	
Self-employed	2,517,246	2827	6.2	5.1–7.6	30.6	28.2–33.1	63.1	60.6–65.6	
Unpaid/homemaker	1,747,038	1979	6.1	4.6–8.1	27.1	24.6–29.7	66.8	63.9–69.6	
Retiree	277,297	360	6.5	3.8–10.9	28.1	22.6–34.2	65.5	59.1–71.3	
***Lifestyle factors***									
**Daily fruit and vegetable intake**									0.04
Adequate	360,253	374	5.6	3.2–9.6	25.4	20.3–31.3	69.0	62.8–74.7	
Inadequate	12,826,531	12,584	8.2	7.5–9.0	31.0	29.9–32.2	60.8	59.5–62.1	
**Alcohol intake**									
Never	11,295,888	11,697	8.1	7.4–8.9	30.5	29.4–31.7	61.3	60.0–62.6	0.36
Ever	1,953,619	1316	8.5	6.5–11.2	33.0	29.7–36.6	58.4	54.5–62.2	
**Smoking**									<0.01
Never	9,988,546	9916	8.0	7.2–8.9	30.0	28.8–31.3	61.9	60.6–63.3	
Current	3,070,519	2910	8.9	7.6–10.4	34.2	31.8–36.7	56.9	54.3–59.5	
Former	197,418	195	5.0	1.7–14.1	24.4	17.7–32.6	70.6	61.6–78.3	
**Physical activity**									<0.0001
Inactive	4,069,283	3775	10.3	9.0–11.8	33.1	31.1–35.1	56.6	54.5–58.7	
Active	9,037,925	9096	7.2	6.4–8.0	29.8	28.5–31.1	63.0	61.6–64.5	

* Perason’s chi-square test was performed. Data source from National Health and Morbidity Survey (NHMS) 2015, B40: bottom 40% (household income below USD 1084); M40: middle 40% (household income between USD 1084 and USD 2452); T20: top 20% (household income above USD 2452).

**Table 3:** Characteristics of the Malaysian adult respondents aged 18 years and above stratified by central adiposity status (*n* = 12,978).

Characteristic	Estimated Population	Count (*n*)	Normal (*n* = 7056)		Obese (*n* = 5922)		*p*-Value *
			Prevalence (%)	95 % CI	Prevalence (%)	95 % CI	
***Sociodemographic***							
**Sex**							<0.0001
Male	6,885,792	6389	67.5	65.8–69.1	32.5	30.9–34.2	
Female	6,310,059	6589	47.8	45.9–49.6	52.2	50.4–54.1	
**Age groups (years)**							<0.0001
18–29	5,156,836	3953	69.5	67.5–71.5	30.5	28.5–32.5	
30–39	3,177,090	2923	53.3	50.8–55.7	46.7	44.3–49.2	
40–49	2,268,503	2460	49.9	47.1–52.7	50.1	47.3–52.9	
50–59	1,513,532	1993	46.5	43.4–49.7	53.5	50.3- 56.6	
≥60	1,079,889	1649	50.7	47.3–54.1	49.3	45.9–52.7	
**Ethnicity**							<0.0001
Malay	7,205,534	8562	57.8	56.3–59.4	42.2	40.6–43.7	
Chinese	3,276,382	2094	63.5	60.5–66.5	36.5	33.5–39.5	
Indian	904,374	907	40.3	35.6–45.2	59.7	54.8–64.4	
Other Bumiputera	1,603,998	1223	56.9	53.2–60.6	43.1	39.4–46.8	
Others	205,563	192	66.1	54.0–76.4	33.9	23.6–46.0	
**Residential area**							0.20
Urban	10,014,803	7408	57.6	56.0–59.3	42.4	40.7–44.0	
Rural	3,181,048	5570	59.4	57.2–61.5	40.6	38.5–42.8	
**Marital status**							<0.0001
Single	4,588,325	3464	70.6	68.6–72.6	29.4	27.4–31.4	
Married	7,915,725	8547	51.8	50.2–53.4	48.2	46.6–49.8	
Widow/widower/divorcee	691,800	967	46.6	42.5–50.8	53.4	49.2–57.5	
**Education level**							<0.01
No formal education	449,409	598	53.7	48.5–58.9	46.3	41.1–51.5	
Primary	1,966,809	2482	53.2	50.4–55.9	46.8	44.1–49.6	
Secondary	6,785,169	6560	58.9	57.2–60.6	41.1	39.4–42.8	
Tertiary	3,865,710	3243	59.2	56.9–61.5	40.8	38.5–43.1	
**Monthly household income**							0.72
B40	8,911,796	9312	57.9	56.4–59.5	42.1	40.5–43.6	
M40	3,352,715	2976	58.8	56.1–61.5	41.2	38.5–43.9	
T20	931,340	690	56.6	51.5–61.6	43.4	38.4–48.5	
**Occupation**							<0.0001
Government	1,376,088	1578	51.4	48.0–54.7	48.6	45.3–52.0	
Private	5,237,209	4174	62.3	60.3–64.2	37.7	35.8–39.7	
Self-employed	2,509,444	2819	60.0	57.4–62.5	40.0	37.5–42.6	
Unpaid/homemaker	1,746,749	1978	41.0	38.0–44.0	59.0	56.0–62.0	
Retiree	280,248	362	52.3	45.5–59.1	47.7	40.9–54.5	
***Lifestyle factors***							
**Daily fruit and vegetable intake**							<0.01
Adequate	355,074	368	48.7	42.6–54.9	51.3	45.1–57.4	
Inadequate	12,771,069	12,545	58.3	56.9–59.6	41.7	40.4–43.1	
**Alcohol intake**							<0.01
Never	11,241,642	11,656	57.3	55.9–58.7	42.7	41.3–44.1	
Ever	1,945,606	1311	62.5	59.0–65.8	37.5	34.2–41.0	
**Smoking**							<0.0001
Never	9,933,316	9880	54.2	52.6–55.8	45.8	44.2–47.4	
Current	3,063,489	2900	70.3	68.0–72.6	29.7	27.4–32.0	
Former	197,418	195	61.8	53.2–69.8	38.2	30.2–46.8	
**Physical activity**							0.53
Inactive	4,060,179	3766	58.5	56.3–60.7	41.5	39.3–43.7	
Active	8,989,194	9063	57.7	56.2–59.3	42.3	40.7–43.8	

* Pearson’s chi-square test was performed. Data source from National Health and Morbidity Survey (NHMS) 2015, B40: Bottom 40% (household income below USD 1084); M40: middle 40% (household income between USD 1084 and USD 2452); T20: top 20% (household income above USD 2452).

## Data Availability

The research data utilized in this present study were retrieved from the NIH Data Repository (https://nihdars.nih.gov.my/, accessed on 19 July 2022) through the request to the Sector for Biostatistics and Data Repository, Office of NIH Manager, National Institutes of Health Malaysia and with permission from the Director General of Ministry of Health Malaysia. All data generated or analyzed during this study are included in this published article and its supplementary information files.

## Supplementary Materials

The following supporting information can be downloaded at: https://www.mdpi.com/article/10.3390/ijerph20043058/s1, Table S1: STROBE Statement—checklist of items that should be included in reports of cross-sectional studies. Table S2: The prevalence of undiagnosed diabetes, undiagnosed hypertension, and undiagnosed hypercholesterolemia stratified by BMI status among the Malaysian adults. Table S3: The prevalence and 95% confidence interval of undiagnosed diabetes, undiagnosed hypertension and undiagnosed hypercholesterolemia stratified by central obesity status among the Malaysian adults. Table S4. Model fit and ROC analysis for final model of multiple logistic regression.

## References

[1] Hruby A., Hu F.B. (2015). The Epidemiology of Obesity: A Big Picture. Pharmacoeconomics.

[2] Albuquerque D., Nóbrega C., Manco L., Padez C. (2017). The contribution of genetics and environment to obesity. Br. Med. Bull..

[3] Beltrán-Carrillo V.J., Megías Á., González-Cutre D., Jiménez-Loaisa A. (2022). Elements behind sedentary lifestyles and unhealthy eating habits in individuals with severe obesity. Int. J. Qual. Stud. Health Well-Being.

[4] World Health Organization (2021). Fact Sheet: Obesity and Overweight.

[5] Mohd-Sidik S., Lekhraj R., Foo C. (2021). Prevalence, Associated Factors and Psychological Determinants of Obesity among Adults in Selangor, Malaysia. Int. J. Environ. Res. Public Health.

[6] Institute of Public Health (IPH) (1996). National Health and Morbidity Survey (NHMS).

[7] Institute of Public Health (IPH) (2019). National Health and Morbidity Survey (NHMS).

[8] Zatońska K., Psikus P., Basiak-Rasała A., Stępnicka Z., Gaweł-Dąbrowska D., Wołyniec M., Gibka J., Szuba A., Połtyn-Zaradna K. (2021). Obesity and Chosen Non-Communicable Diseases in PURE Poland Cohort Study. Int. J. Environ. Res. Public Health.

[9] WHO Expert Consultation (2004). Appropriate body-mass index for Asian populations and its implications for policy and intervention strategies. Lancet.

[10] Institute of Public Health (IPH) (2015). National Health and Morbidity Survey (NHMS).

[11] Sommer I., Teufer B., Szelag M., Nussbaumer-Streit B., Titscher V., Klerings I., Gartlehner G. (2020). The performance of anthropometric tools to determine obesity: A systematic review and meta-analyses. Sci. Rep..

[12] Bhattacharya A., Pal B., Mukherjee S., Roy S.K. (2019). Assessment of nutritional status using anthropometric variables by multivariate analysis. BMC Public Health.

[13] Krishnamoorthy Y., Rajaa S., Murali S., Sahoo J., Kar S.S. (2022). Association Between Anthropometric Risk Factors and Metabolic Syndrome Among Adults in India: A Systematic Review and Meta-Analysis of Observational Studies. Prev. Chronic Dis..

[14] Chen S., Chen Y., Liu X., Li M., Wu B., Li Y., Liang Y., Shao X., Holthofer H., Zou H. (2014). Insulin resistance and metabolic syndrome in normal-weight individuals. Endocrine.

[15] Iacobini C., Pugliese G., Blasetti Fantauzzi C., Federici M., Menini S. (2019). Metabolically healthy versus metabolically unhealthy obesity. Metabolism.

[16] Hopps E., Noto D., Caimi G., Averna M.R. (2010). A novel component of the metabolic syndrome: The oxidative stress. Nutr. Metab. Cardiovasc. Dis..

[17] Kalyan M., Dhore P., Purandare V., Deshpande S., Unnikrishnan A.G. (2020). Obesity and its Link to Undiagnosed Diabetes mellitus Mellitus and High blood pressure in Rural Parts of Western India. Indian J. Endocrinol. Metab..

[18] O’Connor E.A., Evans C.V., Burda B.U., Walsh E.S., Eder M., Lozano P. (2017). Screening for Obesity and Intervention for Weight Management in Children and Adolescents: Evidence Report and Systematic Review for the US Preventive Services Task Force. JAMA.

[19] Luna F., Luyckx V.A. (2020). Why have Non-communicable Diseases been Left Behind?. Asian Bioeth. Rev..

[20] World Health Organization (2000). The Asia-Pacific Perspective: Redefining Obesity and Its Treatment.

[21] Ministry of Health Malaysia (2017). Clinical Practice Guidelines: Management of Dyslipidaemia.

[22] Whelton P.K., Carey R.M., Aronow W.S., Casey D.E., Collins K.J., Himmelfarb C.D., DePalma S.M., Gidding S., Jamerson K.A., Jones D.W. (2018). Guideline for the Prevention, Detection, Evaluation, and Management of High Blood Pressure in Adults: Executive Summary: A Report of the American College of Cardiology/American Heart Association Task Force on Clinical Practice Guidelines. High Blood Press..

[23] Zhang Y.L., Guo S.Q., Ma W.B., Wang J., Bai G.Q., Yang Q., Ti S.F., Ma R., Wei R.P., Liu W.X. (2010). Cut-off points of fasting fingertip capillary blood glucose for detecting both undiagnosed diabetes mellitus and pre-diabetes mellitus. Zhonghua Liu Xing Bing Xue Za Zhi.

[24] National Coordinating Committee on Food and Nutrition (2020). Malaysian Dietary Guidelines.

[25] International Physical Activity Questionnaire (2005). Guidelines for Data Processing and Analysis of the International Physical Activity Questionnaire (IPAQ). www.ipaq.ki.se.

[26] Venkatrao M., Nagarathna R., Majumdar V., Patil S.S., Rathi S., Nagendra H. (2020). Prevalence of Obesity in India and Its Neurological Implications: A Multifactor Analysis of a Nationwide Cross-Sectional Study. Ann. Neurosci..

[27] Haji Zainuddin A.A. (2016). Prevalence and Socio-demographic Determinant of Overweight and Obesity among Malaysian Adult. Int. J. Public Health Res..

[28] Siddiqui M.Z., Donato R. (2016). Overweight and obesity in India: Policy issues from an exploratory multi-level analysis. Health Policy Plan.

[29] Thapa R., Dahl C., Aung W.P., Bjertness E. (2021). Urban-rural differences in overweight and obesity among 25–64 years old Myanmar residents: A cross-sectional, nationwide survey. BMJ Open.

[30] Amanzadeh B., Sokal-Gutierrez K., Barker J.C. (2015). An interpretive study of food, snack and beverage advertisements in rural and urban El Salvador. BMC Public Health.

[31] Hsieh T.-H., Lee J.J., Yu E.W.-R., Hu H.-Y., Lin S.-Y., Ho C.-Y. (2020). Association between obesity and education level among the elderly in Taipei, Taiwan between 2013 and 2015: A cross-sectional study. Sci. Rep..

[32] Bertoia M.L., Mukamal K.J., Cahill L., Hou T., Ludwig D., Mozaffarian D., Willett W.C., Hu F.B., Rimm E.B. (2015). Changes in Intake of Fruits and Vegetables and Weight Change in United States Men and Women Followed for Up to 24 Years: Analysis from Three Prospective Cohort Studies. PLoS Med..

[33] Hebden L., O’Leary F., Rangan A., Lie E.S., Hirani V., Allman-Farinelli M. (2017). Fruit consumption and adiposity status in adults: A systematic review of current evidence. Crit. Rev. Food Sci. Nutr..

[34] Nour M., Lutze S.A., Grech A., Allman-Farinelli M. (2018). The Relationship between Vegetable Intake and Weight Outcomes: A Systematic Review of Cohort Studies. Nutrients.

[35] Tohill B.C., Seymour J., Serdula M., Kettel-Khan L., Rolls B.J. (2004). What epidemiologic studies tell us about the relationship between fruit and vegetable consumption and body weight. Nutr Rev..

[36] Shi Z., Yuan B., Hu G., Dai Y., Zuo H., Holmboe-Ottesen G. (2011). Dietary pattern and weight change in a 5-year follow-up among Chinese adults: Results from the Jiangsu Nutrition Study. Br. J. Nutr..

[37] Rautiainen S., Wang L., Lee I.-M., E Manson J., E Buring J., Sesso H.D. (2015). Higher Intake of Fruit, but Not Vegetables or Fiber, at Baseline Is Associated with Lower Risk of Becoming Overweight or Obese in Middle-Aged and Older Women of Normal BMI at Baseline. J. Nutr..

[38] Yu Z.M., DeClercq V., Cui Y., Forbes C., Grandy S., Keats M., Parker L., Sweeney E., Dummer T.J.B. (2018). Fruit and vegetable intake and body adiposity among populations in Eastern Canada: The Atlantic Partnership for Tomorrow’s Health Study. BMJ Open.

[39] Sarma S., Zaric G.S., Campbell M.K., Gilliland J. (2014). The effect of physical activity on adult obesity: Evidence from the Canadian NPHS panel. Econ. Hum. Biol..

[40] Cook C.M., Schoeller D.A. (2011). Physical activity and weight control. Curr. Opin. Clin. Nutr. Metab. Care.

[41] Fang M., Wang D., Coresh J., Selvin E. (2022). Undiagnosed Diabetes mellitus in U.S. Adults: Prevalence and Trends. Diabetes Mellit. Care.

[42] Lim O.W., Yong C.C. (2019). The Risk Factors for Undiagnosed and Known High blood pressure among Malaysians. Malays J. Med. Sci..

[43] Bushara S.O., Noor S.K., Elmadhoun W.M., Sulaiman A.A., Ahmed M.H. (2015). Undiagnosed high blood pressure in a rural community in Sudan and association with some features of the metabolic syndrome: How serious is the situation?. Ren Fail..

[44] Al-Goblan A.S., A Al-Alfi M., Khan M.Z. (2014). Mechanism linking diabetes mellitus and obesity. Diabetes Metab. Syndr. Obes. Targets Ther..

[45] Li X., Xu J., Yao H., Guo Y., Chen M., Lu W. (2012). Obesity and overweight prevalence and its association with undiagnosed high blood pressure in Shanghai population, China: A cross-sectional population-based survey. Front. Med..

[46] Herman W.H., Ye W., Griffin S.J., Simmons R.K., Davies M.J., Khunti K., Rutten G.E., Sandbaek A., Lauritzen T., Borch-Johnsen K. (2015). Early Detection and Treatment of Type 2 Diabetes mellitus Reduce Cardiovascular Morbidity and Mortality: A Simulation of the Results of the Anglo-Danish-Dutch Study of Intensive Treatment in People with Screen-Detected Diabetes mellitus in Primary Care (ADDITION-Europe). Diabetes Mellit. Care.

